# Untargeted Metabolomic and Lipidomic Profiling Reveals Distinct Biochemical Patterns in Treated Biotinidase Deficiency

**DOI:** 10.3390/ijms27021018

**Published:** 2026-01-20

**Authors:** Ezgi Ünlü Torlak, Merve Koç Yekedüz, Yunus Emre Bülbül, İlknur Sürücü Kara, Sevilay Erdoğan Kablan, Cemil Can Eylem, Büşra Uçar, İncilay Süslü, İpek Baysal, Samiye Yabanoğlu Çiftçi, Fatma Tuba Eminoğlu, Emirhan Nemutlu, Engin Köse

**Affiliations:** 1 Department of Pediatrics, Faculty of Medicine, Ankara University, Ankara 06620, Türkiye; ezgiunnlu@gmail.com (E.Ü.T.); yunusemrebulbul@hotmail.com (Y.E.B.); 2 Division of Pediatric Metabolism, Faculty of Medicine, Ankara University, Ankara 06620, Türkiye; drmervekoc13@hotmail.com (M.K.Y.); drilknursurucu@gmail.com (İ.S.K.); tubaeminoglu@yahoo.com (F.T.E.); 3 Department of Analytical Chemistry, Faculty of Pharmacy, Hacettepe University, Ankara 06230, Türkiye; sevilay.erdogan@hacettepe.edu.tr (S.E.K.); cemilcaneylem@gmail.com (C.C.E.); busra.ucar@hacettepe.edu.tr (B.U.); isuslu@hacettepe.edu.tr (İ.S.); enemutlu@hacettepe.edu.tr (E.N.); 4 Vocational School of Health Services, Hacettepe University, Ankara 06230, Türkiye; ipekbysl@gmail.com; 5 Department of Biochemistry, Faculty of Pharmacy, Hacettepe University, Ankara 06230, Türkiye; samiye@hacettepe.edu.tr; 6 Rare Diseases Application and Research Center, Ankara University, Ankara 06230, Türkiye

**Keywords:** biotinidase deficiency, biotin, metabolomics, lipidomics, octopine, biomarkers, pediatric, mitochondrial metabolism, adaptive metabolism, carboxylase pathways

## Abstract

Biotinidase deficiency is an autosomal recessive disorder that disrupts biotin recycling and multiple carboxylase-dependent pathways. Early and continuous biotin therapy prevents major clinical manifestations, but its long-term biochemical effects remain unclear. This study applied untargeted metabolomic and lipidomic profiling in 54 pediatric patients with genetically confirmed BD receiving regular biotin supplementation and 30 age- and sex-matched controls. Multivariate analyses and pathway enrichment revealed distinct biochemical signatures involving amino acid, energy, and lipid metabolism. Reduced levels of serine, glycine, threonine, and tricarboxylic acid cycle intermediates suggested modified mitochondrial flux, while octopine, exhibiting an approximately 11-fold increase, was the metabolite best able to discriminate between the groups. Lipidomic profiling indicated elevations in sphingolipids, phosphatidylcholines, long-chain fatty acids, and acylcarnitines, consistent with systemic lipid remodeling. These coordinated alterations imply metabolic adaptations to sustained biotin exposure rather than ongoing pathology. Octopine and selected lipid species may represent biochemical indicators of this adaptive state. Overall, the findings highlight that clinically stable children with Biotinidase deficiency exhibit unique metabolic and lipidomic patterns reflecting long-term compensatory mechanisms, underscoring the value of combined omics approaches for understanding disease-specific homeostasis and informing personalized follow-up strategies.

## 1. Introduction

Biotinidase deficiency (BD) is a hereditary metabolic disorder characterized by a defect in the biotinidase enzyme, which is responsible for recycling biotin. It is an autosomal recessive neurocutaneous condition [1]. The global incidence of biotinidase deficiency ranges from approximately 1 in 40,000 to 1 in 60,000; however, higher prevalence rates have been reported in countries with high consanguinity rates, such as Türkiye and Saudi Arabia [2,3]. In Turkey, BD has been included in the national newborn screening program since 2008, with a reported incidence of 1 in 7116, according to the Ministry of Health [4].

Biotinidase liberates biotin during the degradation of biotinylated proteins, enabling its reutilization. Free biotin serves as a cofactor for four key carboxylases, propionyl-CoA carboxylase, 3-methylcrotonyl-CoA carboxylase, pyruvate carboxylase, and acetyl-CoA carboxylase, which play critical roles in gluconeogenesis, branched-chain amino acid catabolism, fatty acid synthesis, and the tricarboxylic acid cycle [5,6,7]. Deficiency of this enzyme results in secondary impairment of these processes, leading to a spectrum of clinical manifestations, such as seizures, hypotonia, developmental delay, and dermatological conditions. Early diagnosis and high-dose biotin supplementation can substantially prevent symptom onset, although neurodevelopmental sequelae may persist in late-diagnosed cases [1,3].

Metabolomic approaches have recently emerged as powerful tools for investigating inborn errors of metabolism, enabling the discovery of novel biochemical signatures and providing insights into disease mechanisms beyond classical markers [8,9]. However, to date, no studies have comprehensively examined the metabolome in patients with BD. Given that BD can influence multiple lipid-related pathways, including fatty acid metabolism, mitochondrial energy production, and membrane remodeling, lipidomic analyses may provide complementary information to capture the broader biochemical context of the disease. The combined use of metabolomics and lipidomics thus offers a systems-level perspective for understanding the long-term biochemical profile of patients receiving continuous biotin supplementation.

The aim of this study was to assess whether children with BD receiving regular biotin therapy (despite being clinically asymptomatic) achieve full biochemical normalization comparable to healthy peers. Using untargeted metabolomic and lipidomic analyses, we investigated whether subtle metabolic differences persist under continuous supplementation, providing a systems-level view of long-term biochemical status in treated patients.

## 2. Results

### 2.1. Demographic and Baseline Characteristics

A total of 54 patients diagnosed with BD and receiving regular biotin supplementation, along with 30 healthy controls, were included in the study. In the patient group, 37% were female and 63% were male, whereas in the control group, 33.3% were female and 66.7% were male. Gender distribution did not significantly differ between groups (*p* = 0.734). The median age of patients was 3.8 years [1.8–6.4], while controls had a median age of 2.5 years [0.7–6.3], with no significant difference (*p* = 0.253). Thus, the two groups were comparable in terms of age and sex (Table 1).

According to biotinidase activity, 16.7% of patients exhibited profound deficiency, 46.3% partial deficiency, and 37% heterozygous levels. The mean baseline biotinidase activity was 1.8 ± 0.9 U/L (0.1–3.8). All patients had initiated biotin supplementation following diagnosis and continued regular treatment. Regarding daily biotin doses, 61.1% of patients received 5 mg/day, 31.5% 10 mg/day, and 7.4% 20 mg/day. The mean enzyme activity at the time of sampling was 2.4 ± 1.2 U/L (0.1–4.7) (Table 1).

Genetic analyses revealed that 31.4% of patients carried compound heterozygous mutations, while 68.6% carried homozygous variants in the BTD gene. The most prevalent genotypes were homozygous p.Asp444His (53.7%), p.Asp444His/p.Arg157His (7.4%), and p.Asp444His/p.Thr532Met (5.5%) (Table 2).

### 2.2. Metabolomic and Lipidomic Profiles

The metabolomic profiles of 54 patients and 30 healthy controls were obtained using GC–MS, which identified 109 metabolites (Table S1). In the same cohort, LC–qTOF–MS-based lipidomics identified 222 lipid species (Table S2).

**Figure 1:**
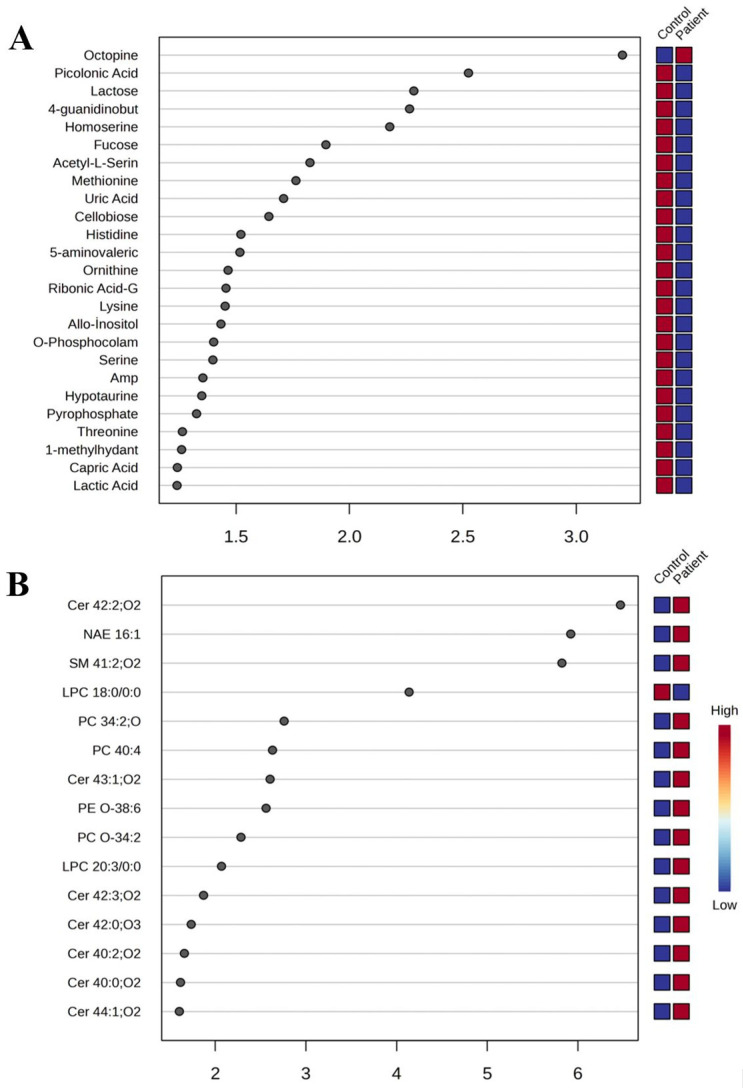
VIP score plots from multivariate analysis comparing pediatric patients with biotinidase deficiency to control groups: (**A**) metabolomics data and (**B**) lipidomics data.

**Figure 2:**
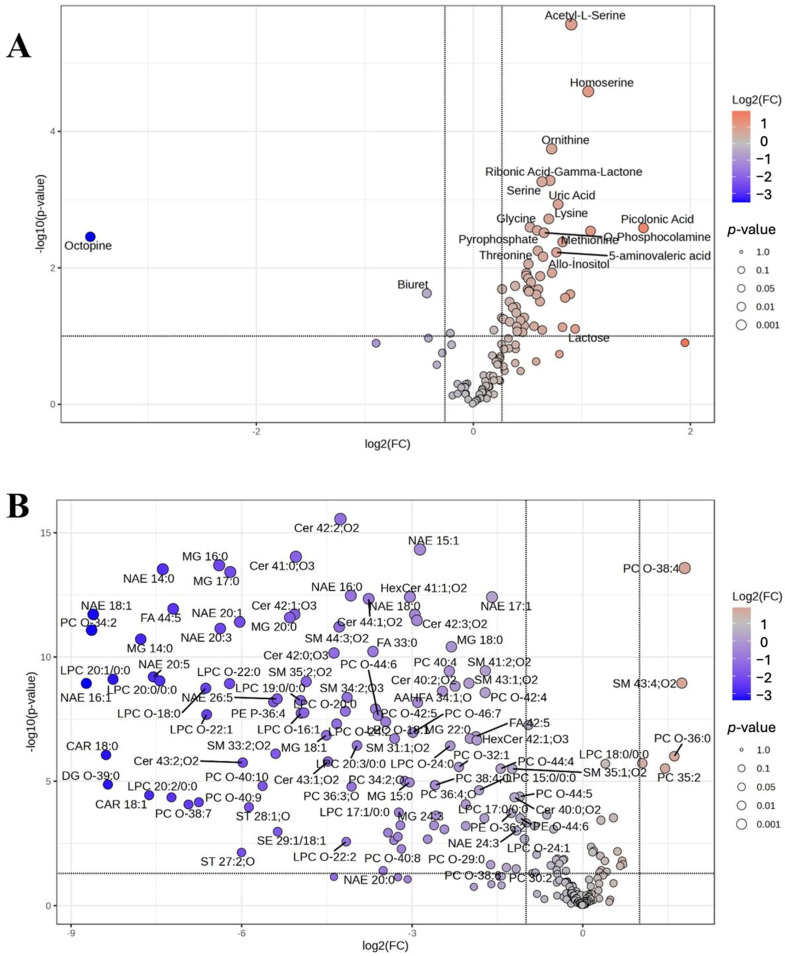
Volcano plots illustrating differential features between pediatric patients with biotinidase deficiency and control groups: (**A**) metabolomics and (**B**) lipidomics data.

Principal component analysis (PCA) was used to visualize global variance structure and detect outliers (Figure S1). One sample in the lipidomics dataset was excluded as an outlier, whereas all metabolomic samples were retained. Partial least squares discriminant analysis (PLS–DA) revealed clear group separation, and variable importance in projection (VIP) scores identified the metabolites and lipids contributing most to class discrimination (Figure 1 and Figure S2). Volcano plot analysis further highlighted statistically significant differences between groups, supporting the presence of distinct metabolic and lipidomic profiles (Figure 2).

#### 2.2.1. Metabolomic Profile Alterations with Biotinidase Deficiency

**Figure 3:**
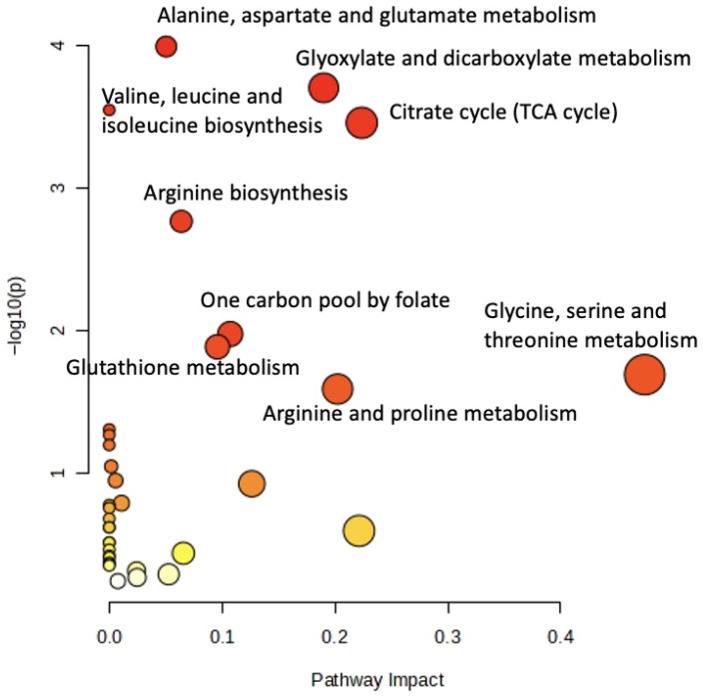
Pathway impact analysis based on metabolomics data from pediatric patients with biotinidase deficiency.

The metabolomic profiles of 54 patients diagnosed with biotinidase deficiency and 30 healthy control subjects were analyzed at the pathway level using GC-MS-based methodologies. Significant metabolic differences were identified between the patient and control groups. The prominent metabolic pathways and related findings are summarized in Figure 3.

##### Amino Acid Metabolism

Significant differences were observed in amino acid metabolism pathways. In patients, the levels of L-alanine, L-asparagine, citrate, fumarate, and 2-oxoglutarate were reduced in the alanine, aspartate, and glutamate metabolism pathway (FDR = 0.007; impact = 0.050). In valine, leucine, and isoleucine biosynthesis, L-threonine, L-isoleucine, and 4-methyl-2-oxopentanoate were lower in patients (FDR = 0.007), with L-threonine being particularly significant (*p* = 0.0011).

In the arginine biosynthesis pathway, reductions were observed in 2-oxoglutarate, fumarate, and L-ornithine among the 14 metabolites analyzed (*p* = 0.0017; FDR = 0.027). L-serine, glycine, and L-threonine were also decreased in glycine, serine, and threonine metabolism (*p* = 0.020; FDR = 0.205). L-serine (*p* = 0.0015) and acetyl-L-serine (*p* < 0.00001) exhibited VIP scores between 1.5 and 2, indicating strong discriminatory potential.

L-proline, L-ornithine, and hydroxyproline were reduced in the arginine and proline metabolism pathway (*p* = 0.025; FDR = 0.230). In glutathione metabolism, glycine, L-ornithine, and 5-oxoproline were reduced (*p* = 0.013; FDR = 0.149). Lysine and 5-aminovaleric acid levels were significantly lower within the lysine degradation pathway (*p* = 0.0001 and *p* = 0.0006, respectively).

##### Energy Metabolism

Twenty metabolites related to the tricarboxylic acid (TCA) cycle were evaluated. 2-Oxoglutarate, isocitrate, citrate, and fumarate levels were lower in patients, although the differences were not statistically significant at the pathway level (*p* = 0.35; FDR = 0.07). In pyruvate metabolism, fumarate and L-lactate were reduced (*p* = 0.064; −log(*p*) = 11.9). These findings suggest subtle differences in mitochondrial-related intermediates.

##### Carbon and Nitrogen Metabolism

Decreased levels of glycine, L-methionine, and L-serine were detected in one-carbon pool metabolism via folate (*p* = 0.01; FDR = 0.142). Although statistical power was limited, these findings indicate biochemical variability that warrants further validation.

In β-alanine metabolism, histidine and uracil were reduced (*p* = 0.05; FDR = 0.395; −log(*p*) = 12.64). Histidine was among the top 25 metabolites based on VIP ranking.

##### Organic Acids and Derivatives

Among organic acids, the metabolite octopine was significantly increased in the patient group, with a VIP score > 3 and *p*~0.003, clearly distinguishing between the two groups. As a result, octopine emerges as a potential candidate biomarker for biotinidase deficiency. In the healthy control group, picolinic acid levels were approximately 2.8-fold higher and supported group differentiation, with a VIP score of approximately 2.5. Additionally, the metabolite homoserine was increased nearly twofold in the healthy control group, exhibiting strong statistical significance with *p*~0.00003 (Figure 1).

##### Other Organic Acids

Several metabolites in glyoxylate and dicarboxylate metabolism were evaluated. 2-Phosphoglycolate, glycine, L-serine, citrate, and isocitrate were significantly decreased in the patient group. Although the *p*-value was 0.20, the calculated values were −log(*p*) = 36.98 and FDR = 0.007.

##### Sugars and Carbohydrate Metabolism

Within the fructose and mannose metabolism pathway, 20 metabolites were analyzed D-mannose levels were lower in the patient group compared with the control group. These differences were statistically supported, with *p* = 0.049 and −log(*p*) = 13.03. Notably, L-fucose ranked among the top 10 metabolites based on VIP scores.

#### 2.2.2. Lipidomic Profile Alterations with Biotinidase Deficiency

Lipidomic profiling revealed significant alterations across multiple lipid classes, including sphingolipids, phospholipids, glycerolipids, fatty acids, acylcarnitines, and sterols. To characterize structural features, fatty acid composition was evaluated based on carbon chain length and degree of unsaturation.

Comparison of lipid profiles between groups demonstrated widespread class-level alterations (Supplementary Figure S2). The most prominently affected classes were sphingolipids, phospholipids, glycerolipids, fatty acids and their derivatives, carnitine derivatives, and sterols (Supplementary Figure S2B).

Within the sphingolipid class, ceramide (Cer) levels were significantly elevated in the patient group; Cer 41:0;O3 and Cer 42:1;O3 metabolites exhibited approximately a 32-fold increase (Supplementary Figure S2B). Other ceramide lipids also demonstrated significant increases, ranging from 7.5- to 64-fold. Among sphingomyelin (SM) species, SM 33:2;O2 showed the most remarkable increase of approximately 42-fold, with other species displaying increases between 10- and 28-fold.

Among phospholipids, the phosphatidylcholine (PC) species PC O-34:2 showed a notable increase of approximately 390-fold in the patient group. Long-chain and polyunsaturated phosphatidylcholine (PC) species were significantly increased in the patient group compared with controls. Within the lysophosphatidylcholine (LPC) subclass, the LPC 20:1/0:0 metabolite increased by approximately 315-fold. Additionally, phosphatidylethanolamine (PE) plasmalogen derivatives were significantly higher in the patient group.

Among glycerolipids, the monoacylglycerol (MG) 14:0 metabolite was increased by approximately 212-fold in the patient group, while diacylglycerol (DG) O-39:0 was approximately 315-fold higher, both demonstrating high statistical significance.

Among fatty acids (FA), FA 44:5 increased by approximately 150-fold, whereas FA 33:0 and FA 42:5 showed 13- and 3.7-fold increases, respectively. Notably, long-chain saturated and polyunsaturated fatty acids were significantly elevated in the patient group.

Among fatty acid derivatives, the N-acylethanolamine (NAE) metabolites NAE 16:1 and NAE 18:1 were approximately 400-fold higher in the patient group, with other NAE species showing increases ranging from 7.5- to 390-fold.

Long-chain acylcarnitines (CARs), a subgroup of carnitine derivatives, were elevated in the patient group, with CAR 18:0, CAR 18:1, and CAR 16:0 increasing by approximately 338-, 200-, and 10-fold, respectively.

Among sterol (ST) lipids, ST 28:0;O and ST 27:2;O metabolites were approximately 60- and 64-fold higher in the patient group. The sterol ester (SE) SE 29:1/18:1 was approximately 43-fold higher in the patient group. No significant differences were observed in cholesterol esters.

## 3. Discussion

This study compared the metabolomic and lipidomic profiles of 54 pediatric patients with biotinidase deficiency receiving regular biotin therapy and 30 age- and sex-matched healthy controls. Using untargeted GC–MS and LC–qTOF–MS analyses, we observed distinct biochemical patterns involving amino acid, energy, and lipid metabolism. These findings suggest that even under continuous biotin supplementation and clinical stability, subtle but consistent biochemical adaptations may occur in treated patients compared with healthy individuals. Among the metabolites most strongly contributing to group separation in PLS–DA, octopine, picolinic acid, acetyl-L-serine, and homoserine showed the highest VIP scores. Rather than diagnostic biomarkers, these metabolites can be considered biochemical indicators that reflect the systemic metabolic state of treated individuals. Notably, octopine was the most consistently altered metabolite across analyses, distinguishing between patient and control groups with high statistical confidence. Multivariate PLS-DA analysis demonstrated clear separation between groups, with high model performance (R^2^, Q^2^), and identified key discriminatory metabolites based on VIP scores (Supplementary Figure S1).

Among the identified metabolites, octopine emerged as the most distinctive, showing the highest VIP score and an approximately 11-fold increase in the patient group. This consistent and statistically significant elevation indicates that octopine is a metabolite of particular interest in the biochemical profile of biotinidase deficiency. In invertebrates, octopine is generated by the NADH-dependent reductive condensation of arginine and pyruvate, catalyzed by octopine dehydrogenase, thereby contributing to NAD⁺ regeneration under anaerobic conditions [10]. Although a dedicated octopine dehydrogenase has not been characterized in humans, low-level production of octopine has been reported in untargeted metabolomics studies, suggesting that analogous reactions may occur via enzyme promiscuity or yet-uncharacterized dehydrogenase activities. In biotinidase deficiency, reduced activity of biotin-dependent carboxylases may alter anaplerotic flux through the tricarboxylic acid (TCA) cycle and shift cytosolic redox balance, potentially favoring alternative NADH-consuming reactions such as the arginine–pyruvate condensation that yields octopine [11]. The observed rise in octopine levels in this study may therefore represent a redox-balancing mechanism associated with chronic metabolic adaptation, rather than ongoing pathology. However, this proposed pathway should be regarded as a mechanistic hypothesis rather than a proven causal relationship, and targeted studies are needed to clarify whether altered carboxylase activity directly contributes to octopine accumulation.

Picolinic acid, the second-highest-ranking metabolite by VIP score, is an intermediate of the kynurenine pathway derived from L-tryptophan metabolism and contributes to NAD⁺ biosynthesis. It is also known to exert neuroprotective and antioxidant effects and to participate in immune modulation under various physiological conditions. In this study, decreased picolinic acid levels were observed in patients with biotinidase deficiency compared with healthy controls. This finding may reflect subtle alterations in tryptophan metabolism and redox-related pathways rather than a direct pathological consequence. Such changes could provide additional insight into the broader biochemical context of biotinidase deficiency and merit further targeted investigation to clarify their mechanistic relevance [12,13].

Lactose, the third most abundant metabolite identified, is a disaccharide involved in carbohydrate metabolism and is hydrolyzed by the enzyme lactase. Lower circulating levels of lactose were observed in patients with biotinidase deficiency compared to controls. While the mechanistic basis of this finding remains uncertain, it may reflect subtle variations in carbohydrate utilization or gut microbial metabolism rather than impaired absorption [14]. Further studies investigating gut–metabolome interactions in this context may help clarify the source and relevance of this observation.

Several metabolites involved in amino acid and energy metabolism showed lower concentrations in patients compared with controls. Decreased 4-guanidinobutyrate may reflect alterations in arginine and creatine pathways, which are known to support nervous system development [15]. Reduced homoserine levels could indicate changes in methionine and threonine biosynthesis [16], while lower fucose may be related to glycosylation and immune-associated pathways [17]. Similarly, decreased acetyl-L-serine may point to shifts in serine metabolism and neurotransmitter precursor balance [18]. Lower methionine levels could influence methylation and antioxidant processes [19,20]. In addition, decreased uric acid may reflect differences in purine metabolism and redox status, whereas reduced cellobiose could be linked to variability in gut microbial carbohydrate metabolism [21].

Biotinidase deficiency involves multiple interconnected biochemical pathways related to energy metabolism, amino acid turnover, and redox balance. In this study, several metabolites—including octopine, picolinic acid, acetyl-L-serine, and homoserine—were identified as key contributors to group differentiation in multivariate analyses. These metabolites provide a biochemical snapshot of systemic metabolic adaptations under continuous biotin therapy rather than direct indicators of dysfunction. The observed alterations in tricarboxylic acid (TCA) cycle intermediates, glutathione metabolism, and amino acid pathways suggest modified mitochondrial-related metabolic fluxes [22,23,24]. While these findings may point to long-term biochemical adjustments in treated individuals, they do not imply ongoing pathology. Instead, they highlight the importance of understanding metabolic adaptations as part of the long-term response to biotin supplementation. Multiple observations collectively support this interpretation. First, all patients were clinically asymptomatic and exhibited normal biochemical parameters, without evidence of acidosis, oxidative stress, or hepatic dysfunction. Second, the directionality of the observed changes (such as elevations in NAEs, phosphatidylcholines, and long-chain acylcarnitines) is consistent with regulated metabolic remodeling rather than canonical markers of mitochondrial dysfunction. Third, alterations tended to occur in a coordinated, pathway-level manner, supporting the notion of controlled metabolic reprogramming. Nevertheless, these signatures should be interpreted as long-term biochemical adaptations with uncertain functional relevance, and subtle subclinical dysfunction cannot be fully excluded.

Lipidomic analyses revealed increased sphingolipid and phospholipid levels in patients compared with controls, suggesting adaptive remodeling of membrane lipids rather than structural disruption. Altered carnitine derivatives indicated enhanced fatty acid oxidation and energy metabolism, while shifts in specific long-chain fatty acids suggested modified lipid turnover. Overall, these findings imply that biotinidase deficiency, even under regular biotin therapy, involves coordinated adjustments in lipid metabolism, warranting further studies to elucidate their mechanistic and adaptive significance [25,26,27].

Although several lipid species exhibited very large fold-changes, these values should be interpreted in the context of their low baseline abundance in controls, such that small absolute increases may yield large numerical fold-changes in untargeted datasets. Notably, the lipid classes showing the greatest increases (such as plasmalogens, N-acylethanolamines (NAEs), and long-chain acylcarnitines) are regulatory molecules involved in membrane remodeling, redox balance, and mitochondrial signaling. Their coordinated elevation therefore suggests an active metabolic reprogramming process rather than passive accumulation due to impaired turnover. Lipid species associated with lipotoxicity or β-oxidation failure were not detected, which further argues against overt pathological disruption, indicating that the observed elevations may reflect adaptive biosynthetic responses to chronic metabolic stress.

Lipidomic profiling showed variable responses among lipid classes, with short-chain N-acylethanolamines (NAEs) displaying the most consistent differences. As bioactive lipids regulating inflammation, redox balance, and cellular homeostasis, NAE changes may reflect adaptive modulation of endocannabinoid-related pathways. These results suggest that long-term biotin therapy is associated with coordinated lipidomic remodeling rather than overt pathological alterations [28].

In contrast, other lipid classes, such as lysophosphatidylcholines (LPCs), monoacylglycerols (MGs), sphingomyelins (SMs), and ether-linked phospholipids, displayed variable alteration patterns, suggesting heterogeneous involvement in lipid remodeling. Ceramides also showed consistent differences, in line with their recognized role in membrane dynamics and cell signaling processes [29]. Collectively, these findings indicate that multiple lipid classes participate in the lipidomic adaptations observed in treated biotinidase deficiency, with the coordinated changes in N-acylethanolamines (NAEs) highlighting their potential role as regulators of metabolic and cellular homeostasis. After FDR correction (FDR < 0.01), a substantial subset of metabolites and lipid species remained significant, and all of the major discriminatory features highlighted in the manuscript (including octopine and key lipid classes) continued to meet statistical significance criteria.

The present study has several limitations. It was conducted at a single center with a relatively small sample size, which may limit the generalizability of the findings. All participants were receiving continuous biotin therapy; therefore, pre-treated and untreated profiles could not be assessed. For ethical reasons, pharmacologic biotin administration to healthy controls or treatment discontinuation in patients was not feasible. Consequently, the biochemical patterns observed reflect the real-world metabolic status of treated individuals rather than controlled pharmacologic exposure. Potential confounders such as diet, microbiota composition, and genetic variability were not fully controlled and may have influenced the metabolomic results. Additionally, biotin doses varied among patients (5–20 mg/day) based on clinical practice, and information on residual biotinidase enzyme activity was not consistently available across participants. As a result, it was not possible to assess dose–response relationships or correlations between metabolite levels, biotin dosage, and residual enzymatic activity. Despite unequal group sizes, the final cohort exceeded the minimum sample size suggested by our a priori power calculation, and key findings remained stable across analyses. While fasting minimized short-term dietary influences, residual dietary variation is inherent to plasma-based lipidomics and may still contribute to variability in lipid profiles. Larger, longitudinal, and mechanistic studies with standardized dosing and repeated omics measurements are warranted to validate and expand these findings.

## 4. Materials and Methods

### 4.1. Sample Collection and Preparation

**Figure 4:**
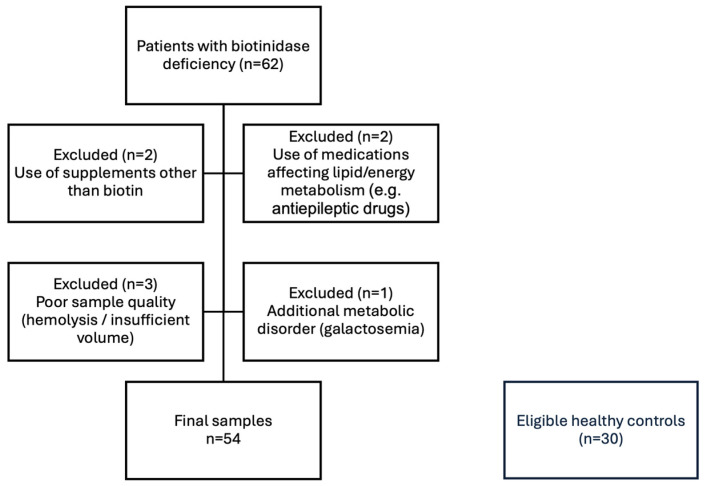
Flowchart illustrating the enrollment, exclusion criteria, and final sample size of pediatric patients with biotinidase deficiency and healthy controls included in the study.

This study was conducted between July 2023 and November 2024 at the Department of Pediatric Metabolic Diseases, Faculty of Medicine, Ankara University. Initially, 62 pediatric patients with genetically confirmed biotinidase deficiency who were receiving regular biotin supplementation were screened for eligibility. Patients using vitamin or micronutrient supplements other than prescribed biotin, patients using medications known to interfere with lipid or energy metabolism, patients with inadequate sample quality (including hemolysis or insufficient plasma volume), and patients with additional metabolic or genetic disorders were excluded. After exclusion criteria were applied, 54 patients were included in the final analysis. A control group of 30 age- and sex-matched healthy children without metabolic disorders and not receiving any chronic medication was also recruited (Figure 4). All participants were clinically stable and free of acute illness at the time of sampling. To minimize short-term dietary variation, fasting venous blood samples (5 mL) were collected from all subjects into EDTA tubes and centrifuged at 3000 rpm for 10 min at +4 °C, and the resulting plasma was aliquoted and stored at −80 °C until analysis. Plasma was selected as the analytical matrix because it is widely used in untargeted lipidomics and allows for systemic profiling of lipid metabolism in a minimally invasive manner in pediatric populations.

### 4.2. Metabolomic and Lipidomic Analyses

Metabolomic and lipidomic profiling was performed using orthogonal analytical platforms: liquid chromatography–quadrupole time-of-flight mass spectrometry (LC-qTOF-MS) and gas chromatography–mass spectrometry (GC-MS). The combined use of these two complementary techniques allowed for broader metabolite coverage. Both metabolomics and lipidomics workflows were adapted from our previous studies [30,31]. A summary of the procedures is provided below.

After plasma samples were thawed at room temperature, 200 µL of each sample was transferred into 2 mL Eppendorf tubes. Then, 400 µL of cold methanol (−20 °C), 400 µL of water, and 400 µL of chloroform were added sequentially. The samples were vortexed for 1 min and shaken at 800 rpm for 20 min. Following centrifugation at 15,000 rpm for 5 min at +4 °C, a three-phase solution was obtained. From the three-phase extraction, the upper polar phase (methanol/water) containing hydrophilic metabolites was collected (200 µL) and stored at −80 °C for subsequent GC-MS analysis, while the lower apolar phase (chloroform) containing lipids was collected (200 µL) and stored at −80 °C for LC-qTOF-MS-based lipidomic analysis.

GC-MS-Based Metabolomics Analysis: Polar fractions were dried to complete dryness in a vacuum concentrator. Samples were derivatized with methoxyamine hydrochloride followed by MSTFA containing 1% TMCS and analyzed via GC-MS using a DB5-MS capillary column. (Agilent J&W Scientific, Santa Clara, CA, USA) Peak separation and retention time alignment were performed using MS-DIAL software. Metabolite identification was achieved by spectral and retention index matching against the Fiehn Retention Index metabolite library.

LC-qTOF-MS-Based Lipidomics Analysis: The apolar fractions were dried under vacuum and reconstituted in 200 µL of isopropanol/acetonitrile (7:3, *v*/*v*). Lipidomic profiling was carried out using LC-qTOF-MS with a C18 column (100 mm × 2.1 mm, 2.7 µm) in both positive and negative electrospray ionization modes. Data processing, including alignment, deconvolution, and retention time correction and annotation, was performed using MS-DIAL software (v5.1). Pooled quality control (QC) plasma samples were analyzed at multiple collision energies (10, 20, and 40 eV), and the obtained MS/MS spectra were searched against spectral libraries for lipid identification.

### 4.3. Data Interpretation and Bioinformatics Analysis

The acquired data were initially evaluated using principal component analysis (PCA) to assess systematic errors and identify outliers. Subsequently, partial least squares discriminant analysis (PLS-DA), based on least squares regression, was applied to determine differences between groups. Discriminatory metabolites were identified using variable importance in projection (VIP) scores, and the direction of significant changes (increase or decrease in specific groups) was interpreted based on regression coefficients. Finally, pathway analysis was conducted to identify the affected metabolic pathways in MetaboAnalyst 6.0.

Patients were classified according to biotinidase enzyme activity as having severe deficiency (<10%), partial deficiency (10–30%), and heterozygous deficiency (30–70%). Although the study was designed with an adaptive approach due to the rarity of the disorder, an a priori power calculation based on effect sizes reported in previous metabolomics studies of rare metabolic diseases (Cohen’s d ≈ 0.7) indicated that a minimum of 25–30 patients and 20 controls would provide >80% power at α = 0.05. Our final cohort of 54 patients and 30 controls therefore exceeded the required sample size. The cutoff value was defined as the mean ± 2 standard deviations (SDs) of the healthy population test results and was used for comparison with the patient groups.

### 4.4. Statistical Analysis

Statistical analyses were performed using IBM SPSS Statistics for Macintosh, version 30.0 (IBM Corp., Armonk, NY, USA). Variables with a normal distribution were expressed as mean ± SD (minimum–maximum), whereas non-normally distributed variables were presented as median [interquartile range (IQR)]. Group comparisons were conducted using the independent-samples *t*-test or Mann–Whitney U test as appropriate. For categorical variables, the Chi-square test or Fisher’s exact test was applied. Tests that are robust to unequal sample sizes (including Welch’s *t*-test when variances were unequal) were applied when necessary to account for the difference in group sizes. Correlation analyses were performed using Pearson’s or Spearman’s correlation coefficients depending on data distribution. To control for potential false positives arising from multiple testing, False Discovery Rate (FDR) correction was applied, with an FDR threshold of <0.01 considered statistically significant. To evaluate multivariate structure and identify discriminatory metabolic features, partial least squares discriminant analysis (PLS-DA) was performed with cross-validation and permutation testing, and variable importance in projection (VIP) scores were calculated. An a priori power calculation indicated that the final cohort size exceeded the minimum sample requirements for detecting moderate to large effect sizes in metabolomics datasets.

## 5. Conclusions

This study offers a comprehensive overview of the metabolic profile of biotinidase deficiency in patients under continuous biotin therapy. Distinct biochemical and lipidomic changes were identified, suggesting that long-term treatment induces measurable metabolic adaptations that differ from patterns in healthy controls. Octopine emerged as the most discriminative metabolite, representing a potential biomarker for further validation. However, whether these alterations stem from the underlying enzyme defect or chronic biotin exposure remains unclear. The results should be viewed as descriptive and hypothesis-generating, underscoring the need for future longitudinal studies to clarify their biological relevance and implications for individualized follow-up.

## Figures and Tables

**Table 1:** Baseline characteristics of patients diagnosed with biotinidase deficiency and healthy controls.

Characteristics	Biotinidase Deficiency (n = 54)	Healthy Control (n = 30)	*p*
Sex F/M, n (%)	20 (37.0)/34 (63.0)	10 (33.3)/20 (66.7)	0.734
Age at Sampling			0.253
Mean (SD) (min–max)	4.5 ± 3.3 (0.3–13.6)	4.0 ± 3.9 (0.1–16.0)	
Median [IQR]	3.8 [1.8–6.4]	2.5 [0.7–6.3]	
Initial Biotinidase Level at Presentation		-	-
Mean (SD) (min–max)	1.8 ± 0.9 (0.1–3.8)		
Median [IQR]	1.8 [1.1–2.4]		
Initial Biotinidase Level Group, n (%)		-	-
Deficiency (<10%)	9 (16.7)		
Partial Deficiency (10–30%)	25 (46.3)		
Heterozygous (30–70%)	20 (37.0)		
Normal (>70%)	0 (0.0)		
Biotin treatment groups, n (%)		-	-
5 mg/day	33 (61.1)		
10 mg/day	17 (31.5)		
20 mg/day	4 (7.4)		
Biotinidase level at the time of metabolomic sampling		-	-
Mean ± SD (min–max)	2.4 ± 1.2 (0.1–4.7)		
Median [IQR]	2.7 [1.4–3.3]		

F: female; IQR: interquartile range; M: male; min–max: minimum–maximum; SD: standard deviation.

**Table 2:** Genetic findings in *BTD* gene in patients diagnosed with biotinidase deficiency.

Allelic Distribution, n (%)	
Homozygous	37 (68.6)
p.Asp444His homozygous	29 (53.7)
p.C33Ffs*36 homozygous	2 (3.7)
p.Arg209Cys homozygous	2 (3.7)
p.Cys13PhefsTer36 homozygous	1 (1.9)
p.Arg157His homozygous	1 (1.9)
p.Arg209His homozygous	1 (1.9)
p.Cys186Tyr homozygous	1 (1.9)
Compound heterozygous	17 (31.4)
p.Asp444His/p.Arg157His	4 (7.4)
p.Asp444His/p.Thr532Met	3 (5.5)
p.Gly14fs*35/p.Cys13Phe	2 (3.7)
p.Asp444His/p.Cys418Ser	1 (1.9)
p.Asp444His/p.C33Ffs*36	1 (1.9)
p.Asp444His/p.Cys13PhefsTer36	1 (1.9)
p.Asp444His/p.Val457Leu	1 (1.9)
p.Arg79Cys/p.Tyr454Cys	1 (1.9)
p.Pro187Ser/p.Pro369Leu	1 (1.9)
p.Asp444His/p.Ala171Thr	1 (1.9)
p.Cys13Phe/p.Ser359Gly	1 (1.9)
Total	54 (100)

## Data Availability

The original contributions presented in this study are included in the article/Supplementary Material. Further inquiries can be directed to the corresponding author.

## Supplementary Materials

The following supporting information can be downloaded at https://www.mdpi.com/article/10.3390/ijms27021018/s1.

## Abbreviations

The following abbreviations are used in this manuscript:

BD	Biotinidase Deficiency
BTD	Biotinidase
WES	Whole-Exome Sequencing
GC–MS	Gas Chromatography–Mass Spectrometry
LC–qTOF–MS	Liquid Chromatography–Quadrupole Time-of-Flight Mass Spectrometry
PCA	Principal Component Analysis
PLS–DA	Partial Least Squares Discriminant Analysis
VIP	Variable Importance in Projection
TCA	Tricarboxylic Acid Cycle
NAE	N-Acylethanolamine
LPC	Lysophosphatidylcholine
PC	Phosphatidylcholine
PE	Phosphatidylethanolamine
SM	Sphingomyelin
Cer	Ceramide
FA	Fatty Acid
CAR	Acylcarnitine
ST	Sterol
SE	Sterol Ester
FDR	False Discovery Rate
QC	Quality Control
EDTA	Ethylenediaminetetraacetic Acid
SD	Standard Deviation
IQR	Interquartile Range
MS	Mass Spectrometry
