# Supplementary material for: Untargeted Metabolomic and Lipidomic Profiling Reveals Distinct Biochemical Patterns in Treated Biotinidase Deficiency
Source: Int J Mol Sci. 2026 Jan 20;27(2):1018. doi: 10.3390/ijms27021018 (PMC12842227; doi:10.3390/ijms27021018)
Supplement: Supplementary file 1 [file ijms-27-01018-s001.zip › Figure S1 S2.pdf]

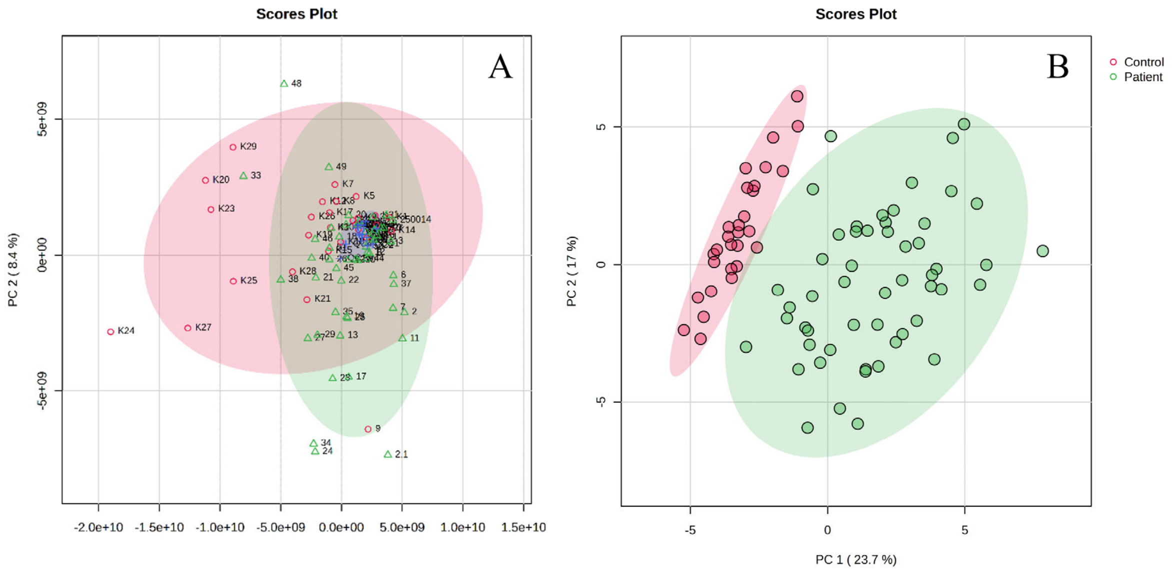

Supplemental Figure S1: PCA score plots illustrating group distribution: (A) metabolomics and (B) lipidomics profiles. Red symbols represent control subjects, while green symbols indicate pediatric patients with biotinidase deficiency. Ellipses correspond to the 95% confidence intervals.

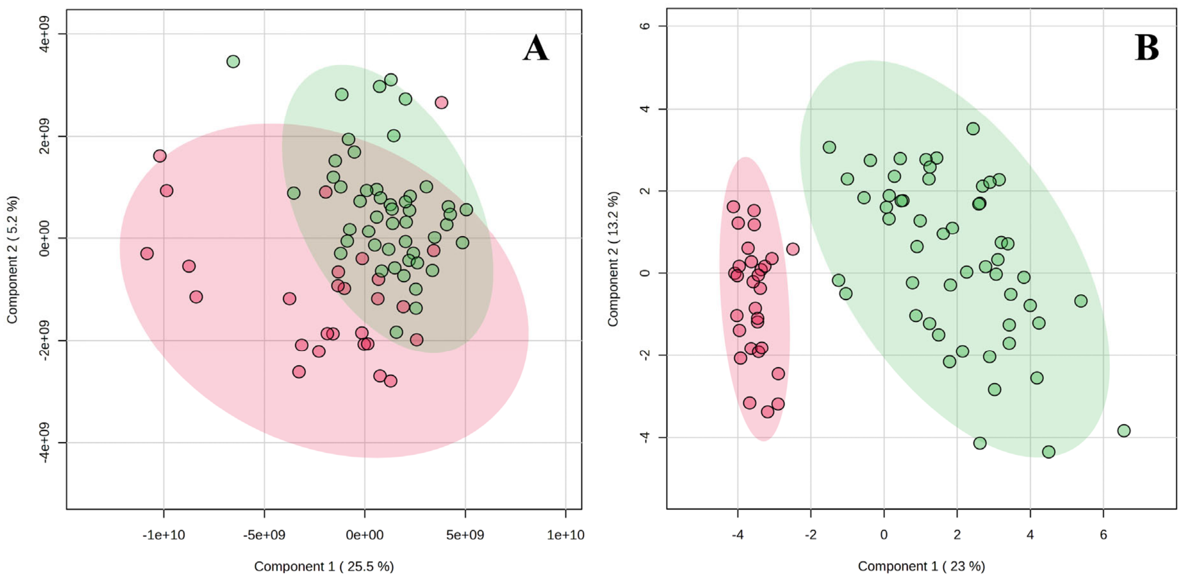

Supplemental Figure S2. PLS-DAscore plots illustrating the separation between study groups: (A) metabolomics and (B) lipidomics profiles. Red circles represent control subjects, while green circles correspond to pediatric patients with biotinidase deficiency. Ellipses indicate the 95% confidence intervals.
