# Supplementary material for: Untargeted Metabolomic and Lipidomic Profiling Reveals Distinct Biochemical Patterns in Treated Biotinidase Deficiency
Source: Int J Mol Sci. 2026 Jan 20;27(2):1018. doi: 10.3390/ijms27021018 (PMC12842227; doi:10.3390/ijms27021018)
Supplement: Supplementary file 1 [file ijms-27-01018-s001.zip › Table S1.pdf]

Table S1. Relative metablolite level

Metabolite name	*Patient*	*Control*
10-hydroxydecanoic acid	1.005 ± 0.899	1.139 ± 0.447
1-methylhydantoin	0.887 ± 0.508	1.39 ± 1.241
2,3-dihydroxybenzoic acid	0.763 ± 0.337	1.345 ± 2.392
2-amino-2-methyl-1,3-propanediol	0.94 ± 0.356	1.211 ± 0.597
2-hydroxybutyric acid	0.987 ± 0.442	1.129 ± 0.889
2-ketoisocaproic acid	0.887 ± 0.369	1.275 ± 0.549
3-phosphoglycerate	1.217 ± 0.843	0.943 ± 0.616
4-guanidinobutyric acid	0.866 ± 1.403	1.438 ± 1.296
4-hydroxy-L-proline	0.858 ± 0.41	1.384 ± 0.968
4-hydroxyquinoline	0.959 ± 0.424	1.172 ± 0.555
5-aminoimidazole-4-carboxamide	0.95 ± 0.066	0.941 ± 0.076
5-aminovaleric acid	0.802 ± 0.363	1.541 ± 1.417
6-hydroxynicotinic acid	0.917 ± 0.131	0.959 ± 0.167
Acetol	0.899 ± 0.6	1.214 ± 0.839
Acetyl-L-serine	0.79 ± 0.348	1.465 ± 0.819
AMP	0.782 ± 1.008	1.551 ± 2.297
Alanine	0.952 ± 0.223	1.089 ± 0.251
Allo-inositol	0.864 ± 0.384	1.383 ± 0.936
Allose	0.887 ± 0.522	1.239 ± 0.725
Alpha ketoglutaric acid	0.821 ± 0.524	1.426 ± 0.994
Ascorbic acid	0.913 ± 0.529	1.223 ± 0.716
Asparagine	0.934 ± 0.168	1.138 ± 0.283
Aspartic acid	0.916 ± 1.398	1.037 ± 1.14
Beta- alanine	0.341 ± 1.99	0.805 ± 2.62
Beta-glycerolphosphate	0.961 ± 0.366	1.039 ± 0.323
Biuret	1.135 ± 0.632	0.831 ± 0.374
Capric acid	0.819 ± 0.453	1.257 ± 0.862
Cellobiose	0.744 ± 1.367	1.615 ± 2.985
Cholesterol	1 ± 0.174	1.057 ± 0.195
Citric acid	0.905 ± 0.398	1.236 ± 0.711
Citrulline	0.948 ± 0.618	1.197 ± 0.804
Creatinine	1.058 ± 0.397	0.994 ± 0.411
Cycloleucine	1.01 ± 0.744	1.011 ± 0.605
Erythrose-4-Phosphate	0.85 ± 2.389	1.64 ± 6.006
Farnesal	0.884 ± 0.603	1.217 ± 0.911
Fucose	0.849 ± 0.568	1.38 ± 1.648
Fumaric acid	0.94 ± 0.21	1.143 ± 0.26
Galactose	0.9 ± 0.397	1.16 ± 0.939
Gluconic acid lactone	0.876 ± 0.483	1.139 ± 0.265
Glutamic acid	0.877 ± 0.202	0.898 ± 0.299
Glutamine	0.999 ± 0.629	1.246 ± 0.768
Glyceraldehyde	1.129 ± 0.66	1.005 ± 0.846
Glyceric acid	1.082 ± 0.41	0.905 ± 0.679
Glycerol	1.042 ± 0.431	1.07 ± 0.472

Glycerol 1-phosphate	0.958 ± 0.524	1.004 ± 0.367
Glycine	0.883 ± 0.338	1.3 ± 0.757
Glycolic acid	0.916 ± 0.413	0.964 ± 0.541
Heptadecanoic acid	0.924 ± 0.362	1.102 ± 0.701
Histidine	0.884 ± 0.625	1.437 ± 1.19
Homoserine	0.841 ± 0.448	1.477 ± 1.002
Hydroxyurea	0.873 ± 0.333	1.143 ± 0.439
Hypotaurine	0.873 ± 1.387	1.463 ± 2.116
Iminodiacetic acid	0.931 ± 0.278	1.131 ± 0.356
Isocitric acid	0.864 ± 0.498	1.31 ± 0.745
Isoleucine	0.863 ± 0.388	1.411 ± 1.06
Itaconic acid	0.969 ± 0.204	1.144 ± 0.197
Lactamide	1.046 ± 0.791	1.037 ± 0.502
Lactic acid	0.841 ± 0.639	1.334 ± 1.328
Lactose	0.828 ± 1.205	1.496 ± 2.209
Lauric acid	0.924 ± 0.39	1.231 ± 0.884
Linoleic acid	0.955 ± 0.565	1.046 ± 0.429
Lysine	0.83 ± 0.363	1.434 ± 0.926
Lyxose	0.948 ± 1.643	1.033 ± 1.11
Malic acid	1.047 ± 1.668	1.248 ± 1.529
Malonic acid	0.907 ± 0.599	1.162 ± 0.672
Mannose	0.928 ± 0.45	1.167 ± 0.568
Methionine	0.784 ± 0.534	1.632 ± 1.59
Methyl laurate	0.941 ± 0.868	1.187 ± 1.016
Methyl Myristate	1.357 ± 2.372	0.552 ± 0.792
Methyl-Beta-D-Galactopgranoside	0.922 ± 0.812	1.184 ± 0.889
Muconic Acid	0.835 ± 0.244	1.216 ± 2.257
Myristic Acid	0.887 ± 0.55	1.106 ± 0.812
N-(2-Hydroxyethyl)iminodiacetic Acid	1.008 ± 0.027	0.978 ± 0.086
N-Acetyl-L-aspartic acid	0.988 ± 0.095	1.033 ± 0.176
Octopine	1.541 ± 2.118	0.385 ± 1.519
Oleic acid	0.931 ± 0.407	1.082 ± 0.359
O-Phosphocolamine	0.89 ± 0.439	1.36 ± 0.81
Ornithine	0.833 ± 0.444	1.472 ± 0.933
Oxalacetic acid	0.871 ± 0.557	1.521 ± 2.41
Oxalic acid	0.958 ± 0.339	0.959 ± 0.537
Palmitic acid	0.933 ± 0.31	1.001 ± 0.352
Palmitoleic acid	1.011 ± 1.023	0.912 ± 0.574
Phenylacetaldehyde	1.055 ± 0.904	1.063 ± 0.468
Phenylalanine	0.955 ± 0.367	1.138 ± 0.486
Phosphoglycolic acid	0.84 ± 0.423	1.339 ± 0.774
Picolonic acid	0.575 ± 0.809	1.408 ± 1.889
Pipecolic acid	0.937 ± 0.381	1.139 ± 0.566
Porphine	0.963 ± 0.217	1.1 ± 0.343
Proline	0.92 ± 0.484	1.21 ± 0.672
Pyroglutamic acid	0.894 ± 0.399	1.277 ± 0.715

Pyrophosphate	0.945 ± 0.5	1.287 ± 0.56
Ribitol	0.866 ± 0.589	1.264 ± 0.966
Sarcosine	1.042 ± 0.876	1.065 ± 0.442
Serine	0.894 ± 0.393	1.298 ± 0.711
Stearic acid	0.953 ± 0.192	0.993 ± 0.25
Succinic acid	0.985 ± 0.104	0.947 ± 0.132
Sucrose	0.783 ± 0.423	1.521 ± 3.503
Tartaric Acid	0.976 ± 0.353	1.054 ± 0.315
Threitol	0.902 ± 0.367	1.283 ± 0.592
Threo-Beta-hydroxyaspartic acid	1.105 ± 0.486	1.021 ± 0.461
Threonine	0.876 ± 0.386	1.341 ± 0.843
Threose	0.927 ± 0.322	1.087 ± 0.212
Thymol	0.969 ± 0.129	0.99 ± 0.168
Tyrosine methyl ester	0.932 ± 0.191	1.111 ± 0.336
Uracil	0.902 ± 0.428	1.314 ± 0.882
Urea	1.031 ± 0.386	0.953 ± 0.397
Uric acid	0.865 ± 0.388	1.379 ± 0.94
Valine	0.96 ± 0.63	1.302 ± 0.96
Xylitol	0.926 ± 1.534	1.494 ± 3.28
