# Supplementary material for: Untargeted Metabolomic and Lipidomic Profiling Reveals Distinct Biochemical Patterns in Treated Biotinidase Deficiency
Source: Int J Mol Sci. 2026 Jan 20;27(2):1018. doi: 10.3390/ijms27021018 (PMC12842227; doi:10.3390/ijms27021018)
Supplement: Supplementary file 1 [file ijms-27-01018-s001.zip › Table S2.pdf]

Table S2. Group-wise lipid levels and p-values from Student's t-test

Lipid	*Control*	*Patient*	*P Values*
AAHFA 34:1;O	0.038±0.018	0.212±0.016	0.000
CAR 14:1	0.057±0.015	0.047±0.008	0.515
CAR 16:0	0.020±0.009	0.198±0.041	0.002
CAR 18:0	0.000±0.000	0.133±0.018	0.000
CAR 18:1	0.002±0.000	0.306±0.051	0.000
CAR 18:2	0.222±0.032	0.270±0.059	0.562
CE 18:0	0.004±0.001	0.012±0.003	0.068
Cer 40:0;O2	0.456±0.028	1.050±0.100	0.000
Cer 40:0;O3	0.019±0.007	0.034±0.01	0.338
Cer 40:2;O2	0.163±0.062	0.772±0.057	0.000
Cer 41:0;O2	0.682±0.046	0.69±0.042	0.904
Cer 41:0;O3	0.006±0.004	0.202±0.015	0.000
Cer 42:0;O2	2.516±0.171	2.100±0.145	0.079
Cer 42:0;O3	0.032±0.028	0.669±0.061	0.000
Cer 42:1;O3	0.005±0.003	0.178±0.015	0.000
Cer 42:2;O2	0.131±0.076	2.507±0.165	0.000
Cer 42:3;O2	0.105±0.053	0.792±0.055	0.000
Cer 42:3;O3	0.001±0.000	0.003±0.001	0.137
Cer 43:1;O2	0.045±0.01	1.002±0.136	0.000
Cer 43:2;O2	0.004±0.001	0.264±0.037	0.000
Cer 44:1;O2	0.047±0.005	0.636±0.05	0.000
Cer 50:7;O4	0.100±0.019	0.148±0.011	0.024
DG O-37:1	0.002±0.000	0.015±0.006	0.087
DG O-39:0	0.001±0.000	0.37±0.059	0.000
FA 12:0	0.239±0.050	0.21±0.03	0.604
FA 14:0	1.860±0.217	1.739±0.127	0.606
FA 14:1	0.276±0.041	0.379±0.042	0.116
FA 15:0	0.287±0.021	0.261±0.009	0.168
FA 16:0	0.493±0.017	0.431±0.014	0.006
FA 16:3	0.136±0.013	0.186±0.017	0.053
FA 17:0	0.393±0.046	0.373±0.022	0.665
FA 18:0	0.543±0.016	0.482±0.014	0.007
FA 18:1;O	0.118±0.017	0.212±0.017	0.001
FA 18:3	0.522±0.068	0.475±0.041	0.532
FA 20:0	1.143±0.020	1.033±0.026	0.004
FA 20:1	0.746±0.054	0.77±0.059	0.786
FA 20:2	0.615±0.058	0.745±0.049	0.107
FA 20:3	0.324±0.025	0.502±0.031	0.000
FA 21:0	0.130±0.004	0.12±0.004	0.089
FA 22:1	0.12±0.007	0.191±0.087	0.548
FA 22:4	0.573±0.051	0.819±0.044	0.001
FA 22:5	0.257±0.024	0.355±0.026	0.014
FA 24:0	0.209±0.015	0.168±0.008	0.010

FA 28:7	0.316±0.01	0.24±0.01	0.000
FA 33:0	0.008±0.001	0.106±0.01	0.000
FA 40:5	0.365±0.011	0.349±0.011	0.374
FA 42:5	0.191±0.072	0.702±0.053	0.000
FA 44:5	0.001±0.000	0.16±0.014	0.000
Hex2Cer 34:1;O2	0.187±0.013	0.211±0.011	0.170
Hex2Cer 42:2;O2	0.081±0.009	0.081±0.007	0.947
HexCer 41:1;O2	0.062±0.029	0.511±0.035	0.000
HexCer 42:1;O3	0.064±0.019	0.232±0.019	0.000
LPC 14:0/0:0	0.114±0.025	0.144±0.026	0.453
LPC 15:0/0:0	0.030±0.008	0.106±0.012	0.000
LPC 16:0	1.663±0.129	1.588±0.1	0.653
LPC 16:1	0.157±0.02	0.168±0.021	0.729
LPC 17:0/0:0	0.107±0.037	0.445±0.057	0.000
LPC 17:1/0:0	0.002±0.001	0.023±0.004	0.000
LPC 18:0	1.192±0.072	1.098±0.061	0.342
LPC 18:0/0:0	2.950±0.152	1.429±0.202	0.000
LPC 18:1/0:0	0.678±0.083	0.485±0.246	0.571
LPC 18:3/0:0	0.015±0.011	0.011±0.006	0.711
LPC 19:0/0:0	0.003±0.002	0.085±0.009	0.000
LPC 20:0/0:0	0.001±0.000	0.13±0.014	0.000
LPC 20:1/0:0	0.001±0.000	0.179±0.019	0.000
LPC 20:2/0:0	0.001±0.000	0.108±0.018	0.000
LPC 20:3	0.216±0.037	0.2±0.023	0.695
LPC 20:3/0:0	0.052±0.02	0.812±0.1	0.000
LPC 20:4	1.132±0.136	1.101±0.112	0.864
LPC 20:5/0:0	0.03±0.006	0.034±0.017	0.844
LPC 22:0/0:0	0.001±0.000	0.011±0.002	0.001
LPC 22:4/0:0	0.001±0.000	0.003±0.001	0.153
LPC 22:5/0:0	0.005±0.001	0.033±0.007	0.002
LPC 22:6	0.264±0.034	0.163±0.023	0.016
LPC 22:6/0:0	0.590±0.101	0.601±0.092	0.942
LPC 24:0/0:0	0.002±0.000	0.039±0.004	0.000
LPC O-16:1	0.007±0.003	0.227±0.026	0.000
LPC O-18:0	0.002±0.001	0.214±0.023	0.000
LPC O-18:1	0.035±0.008	0.385±0.042	0.000
LPC O-20:0	0.001±0.001	0.041±0.005	0.000
LPC O-20:1	0.001±0.000	0.007±0.002	0.071
LPC O-22:0	0.001±0.000	0.061±0.006	0.000
LPC O-22:1	0.000±0.000	0.041±0.005	0.000
LPC O-22:2	0.000±0.000	0.002±0.001	0.003
LPC O-24:0	0.017±0.007	0.085±0.008	0.000
LPC O-24:1	0.051±0.012	0.103±0.01	0.002
LPC O-24:2	0.002±0.001	0.045±0.005	0.000
LPE 18:0	0.228±0.014	0.234±0.015	0.782
LPE 18:1	0.22±0.045	0.199±0.022	0.648

LPE 18:2	0.189±0.033	0.232±0.026	0.320
MG 14:0	0.001±0.000	0.186±0.017	0.000
MG 15:0	0.001±0.000	0.005±0.001	0.000
MG 16:0	0.004±0.001	0.373±0.029	0.000
MG 17:0	0.001±0.000	0.091±0.007	0.000
MG 18:0	0.062±0.006	0.309±0.024	0.000
MG 18:1	0.004±0.001	0.093±0.011	0.000
MG 18:2	0.002±0.000	0.018±0.004	0.002
MG 20:0	0.013±0.001	0.447±0.039	0.000
MG 22:0	0.011±0.004	0.043±0.003	0.000
MG 24:3	0.026±0.008	0.154±0.024	0.000
MG 26:4	0.005±0.001	0.014±0.003	0.066
MG 37:1	0.001±0.000	0.013±0.005	0.069
NAE 14:0	0.002±0.000	0.382±0.03	0.000
NAE 15:1	0.032±0.004	0.234±0.015	0.000
NAE 16:0	0.014±0.001	0.228±0.018	0.000
NAE 16:1	0.005±0.000	2.18±0.233	0.000
NAE 17:0	2.481±0.057	2.539±0.036	0.370
NAE 17:1	0.068±0.003	0.207±0.012	0.000
NAE 18:0	0.02±0.003	0.154±0.012	0.000
NAE 18:1	0.001±0.0	0.363±0.032	0.000
NAE 19:2	0.096±0.016	0.108±0.007	0.450
NAE 20:0	0.000±0.000	0.005±0.002	0.039
NAE 20:1	0.004±0.000	0.239±0.021	0.000
NAE 20:2	0.004±0.001	0.007±0.001	0.009
NAE 20:3	0.001±0.000	0.044±0.004	0.000
NAE 20:5	0.001±0.000	0.148±0.015	0.000
NAE 22:4	0.003±0.001	0.002±0.0	0.022
NAE 24:3	0.004±0.001	0.01±0.001	0.001
NAE 26:5	0.001±0.000	0.058±0.006	0.000
PC 26:0	0.041±0.014	0.056±0.011	0.397
PC 30:2	0.032±0.014	0.073±0.011	0.033
PC 32:2	1.303±0.145	1.774±0.112	0.013
PC 33:1	0.466±0.05	0.534±0.041	0.315
PC 33:2	0.635±0.07	0.725±0.041	0.241
PC 34:2	0.003±0.0	0.015±0.003	0.001
PC 34:2;O	0.131±0.023	1.144±0.158	0.000
PC 34:3	2.086±0.22	2.545±0.17	0.108
PC 34:4	0.329±0.039	0.452±0.037	0.037
PC 35:1	0.791±0.062	0.85±0.057	0.514
PC 35:2	0.09±0.011	0.033±0.006	0.000
PC 35:4	0.232±0.021	0.258±0.019	0.387
PC 36:3;O	0.011±0.003	0.187±0.028	0.000
PC 36:4;O	0.092±0.017	0.558±0.074	0.000
PC 36:4	0.15±0.025	0.566±0.224	0.176
PC 38:2	1.253±0.122	1.123±0.075	0.343

PC 38:4;O	0.034±0.007	0.204±0.027	0.000
PC 38:4	2.11±0.236	2.282±0.131	0.491
PC 38:6	2.041±0.164	2.6±0.139	0.015
PC 40:4	0.236±0.018	1.203±0.099	0.000
PC 40:5	1.018±0.081	0.701±0.043	0.000
PC 40:7	0.559±0.054	0.447±0.026	0.037
PC 40:8	0.374±0.03	0.368±0.022	0.883
PC O-29:0	0.037±0.016	0.115±0.023	0.023
PC O-31:0	0.418±0.034	0.513±0.028	0.040
PC O-32:1	0.017±0.005	0.077±0.008	0.000
PC O-34:2	0.002±0.0	0.841±0.077	0.000
PC O-34:3	0.013±0.002	0.024±0.004	0.050
PC O-35:3;O3	0.18±0.017	0.192±0.032	0.793
PC O-36:0	0.059±0.007	0.019±0.004	0.000
PC O-36:3	0.348±0.036	0.689±0.066	0.000
PC O-36:5	0.01±0.002	0.007±0.001	0.126
PC O-38:4	0.518±0.029	0.15±0.025	0.000
PC O-38:5	2.835±0.127	3.017±0.123	0.345
PC O-38:6	0.102±0.006	0.259±0.052	0.029
PC O-38:7	0.001±0.0	0.073±0.013	0.000
PC O-40:10	0.0±0.0	0.006±0.001	0.000
PC O-40:4	0.166±0.014	0.322±0.018	0.000
PC O-40:5	0.394±0.02	0.425±0.018	0.267
PC O-40:6	0.402±0.029	0.392±0.026	0.801
PC O-40:7	0.463±0.054	0.361±0.027	0.061
PC O-40:8	0.002±0.0	0.017±0.004	0.005
PC O-40:9	0.002±0.001	0.217±0.038	0.000
PC O-42:2	0.171±0.02	0.201±0.012	0.173
PC O-42:4	0.072±0.02	0.235±0.015	0.000
PC O-42:5	0.038±0.005	0.288±0.028	0.000
PC O-42:6	0.174±0.014	0.219±0.011	0.014
PC O-44:4	0.156±0.039	0.424±0.033	0.000
PC O-44:5	0.247±0.05	0.532±0.04	0.000
PC O-44:6	0.019±0.006	0.232±0.025	0.000
PC O-46:7	0.011±0.007	0.085±0.009	0.000
PC O-48:4	0.001±0.0	0.005±0.001	0.001
PE 34:1	0.207±0.019	0.239±0.021	0.315
PE 34:2	0.55±0.046	0.682±0.056	0.117
PE 36:1	0.345±0.055	0.382±0.039	0.579
PE 36:2	1.491±0.147	1.685±0.147	0.393
PE 36:3	0.557±0.058	0.582±0.055	0.781
PE 36:4	0.48±0.067	0.526±0.056	0.608
PE 38:3	0.216±0.015	0.218±0.014	0.950
PE 38:6	0.45±0.064	0.372±0.035	0.245
PE O-34:2	0.373±0.035	0.345±0.019	0.450
PE O-34:3	0.673±0.04	0.762±0.041	0.159

PE O-36:2	0.113±0.032	0.269±0.024	0.000
PE O-37:5	0.079±0.012	0.141±0.022	0.047
PE O-38:6	0.405±0.175	1.346±0.158	0.000
PE O-38:7	0.142±0.025	0.137±0.019	0.886
PE O-40:6	0.219±0.024	0.271±0.02	0.112
PE O-40:8	1.113±0.186	0.705±0.085	0.025
PE O-44:6	0.029±0.006	0.062±0.005	0.000
PE P-36:3	0.004±0.003	0.017±0.002	0.001
PE P-36:4	0.005±0.001	0.238±0.026	0.000
PI 38:4	0.234±0.016	0.224±0.011	0.629
SE 29:1/18:1	0.0±0.0	0.019±0.004	0.001
SM 31:1;O2	0.009±0.004	0.092±0.01	0.000
SM 32:0;O2	0.217±0.022	0.178±0.015	0.130
SM 32:2;O2	0.276±0.024	0.291±0.021	0.671
SM 33:1;O2	2.449±0.147	2.369±0.121	0.685
SM 33:2;O2	0.001±0.0	0.061±0.008	0.000
SM 34:0;O2	0.848±0.035	0.68±0.028	0.000
SM 34:0;O3	0.152±0.007	0.155±0.008	0.802
SM 34:1;O3	0.272±0.017	0.254±0.014	0.419
SM 34:2;O3	0.004±0.003	0.066±0.007	0.000
SM 35:1;O2	0.071±0.006	0.168±0.014	0.000
SM 35:2;O2	0.006±0.001	0.169±0.017	0.000
SM 36:0;O2	0.382±0.045	0.25±0.025	0.007
SM 36:2;O2	1.705±0.082	1.631±0.073	0.527
SM 38:0;O2	0.713±0.051	0.544±0.042	0.016
SM 39:1;O2	2.177±0.222	2.436±0.141	0.308
SM 40:0;O2	1.687±0.081	1.375±0.105	0.046
SM 40:1;O2	0.099±0.011	0.074±0.007	0.043
SM 41:2;O2	0.939±0.073	3.078±0.217	0.000
SM 41:3;O2	0.163±0.016	0.156±0.017	0.767
SM 42:0;O2	0.272±0.015	0.253±0.013	0.358
SM 43:1;O2	0.092±0.026	0.368±0.026	0.000
SM 43:2;O2	0.521±0.084	0.693±0.059	0.096
SM 43:3;O2	0.21±0.025	0.279±0.017	0.020
SM 43:4;O2	0.127±0.007	0.038±0.009	0.000
SM 44:1;O2	0.02±0.004	0.255±0.027	0.000
SM 44:2;O2	0.006±0.001	0.053±0.01	0.001
SM 44:3;O2	0.006±0.005	0.114±0.01	0.000
ST 27:1;O;S	0.194±0.015	0.189±0.011	0.780
ST 27:2;O	0.001±0.0	0.087±0.023	0.007
ST 28:1;O	0.0±0.0	0.012±0.002	0.000
